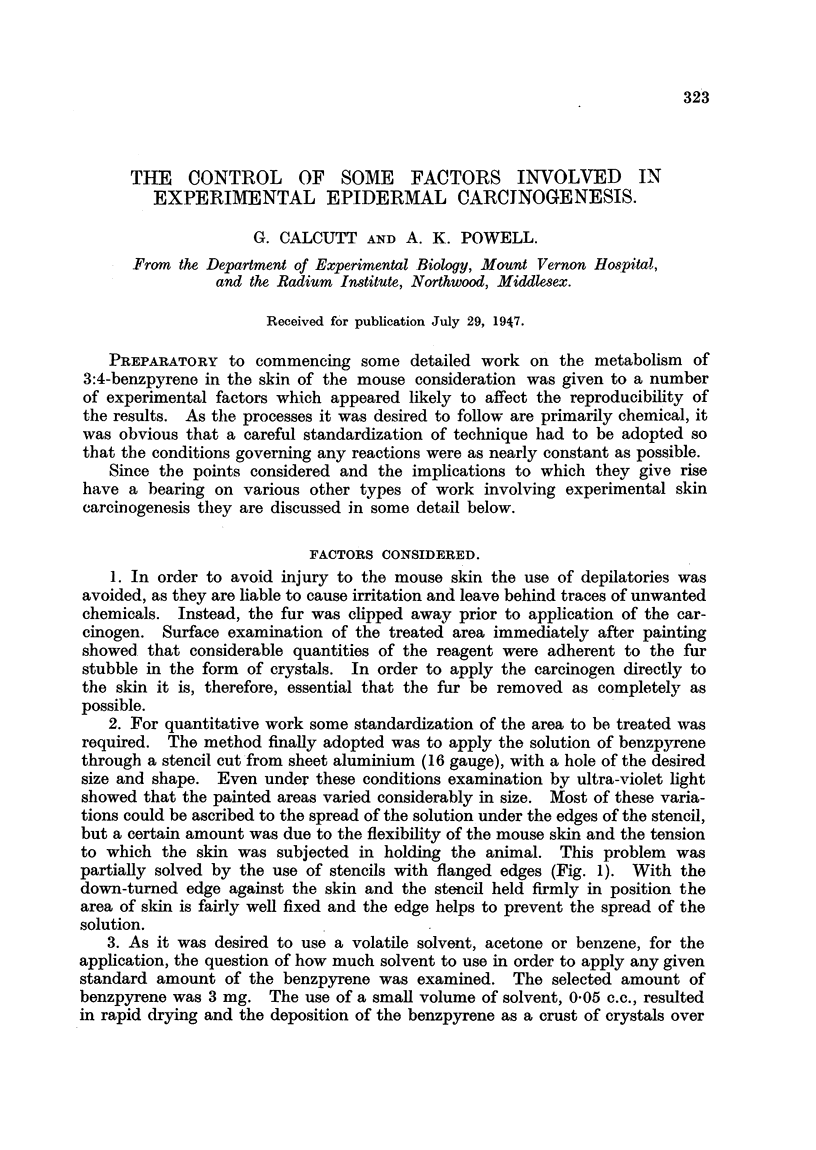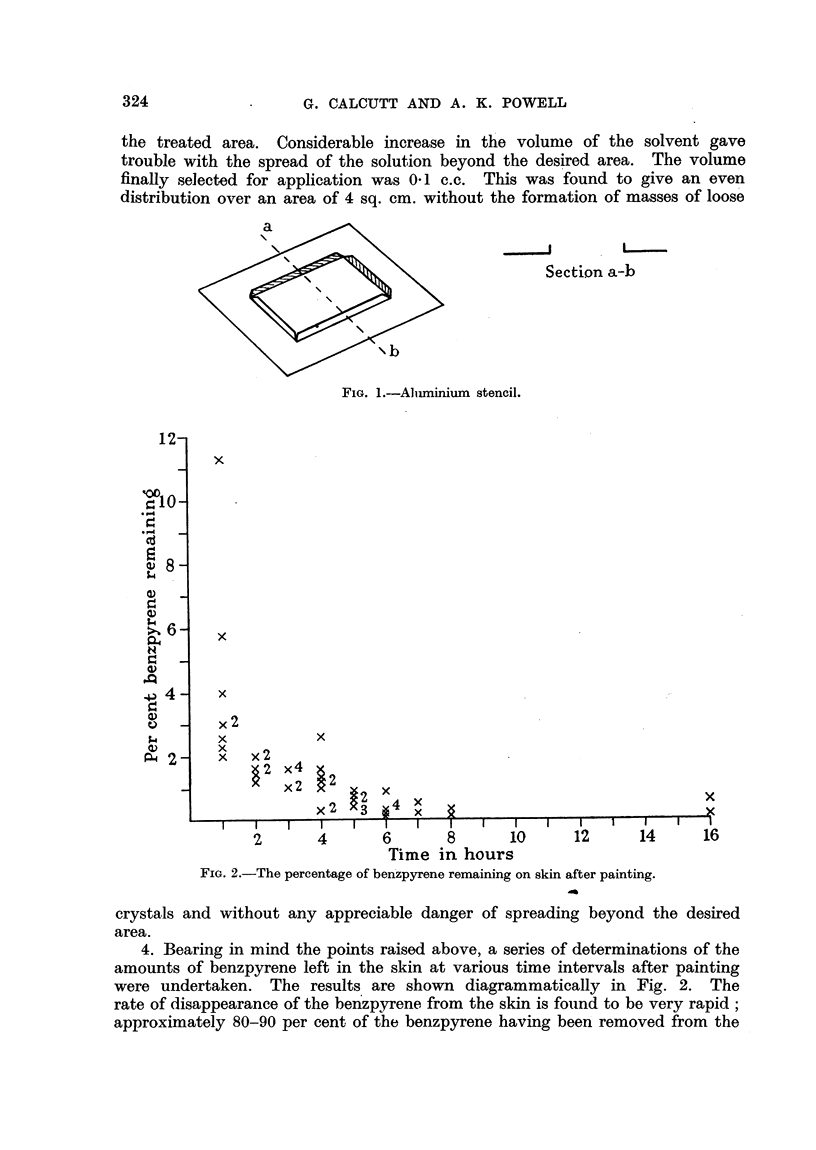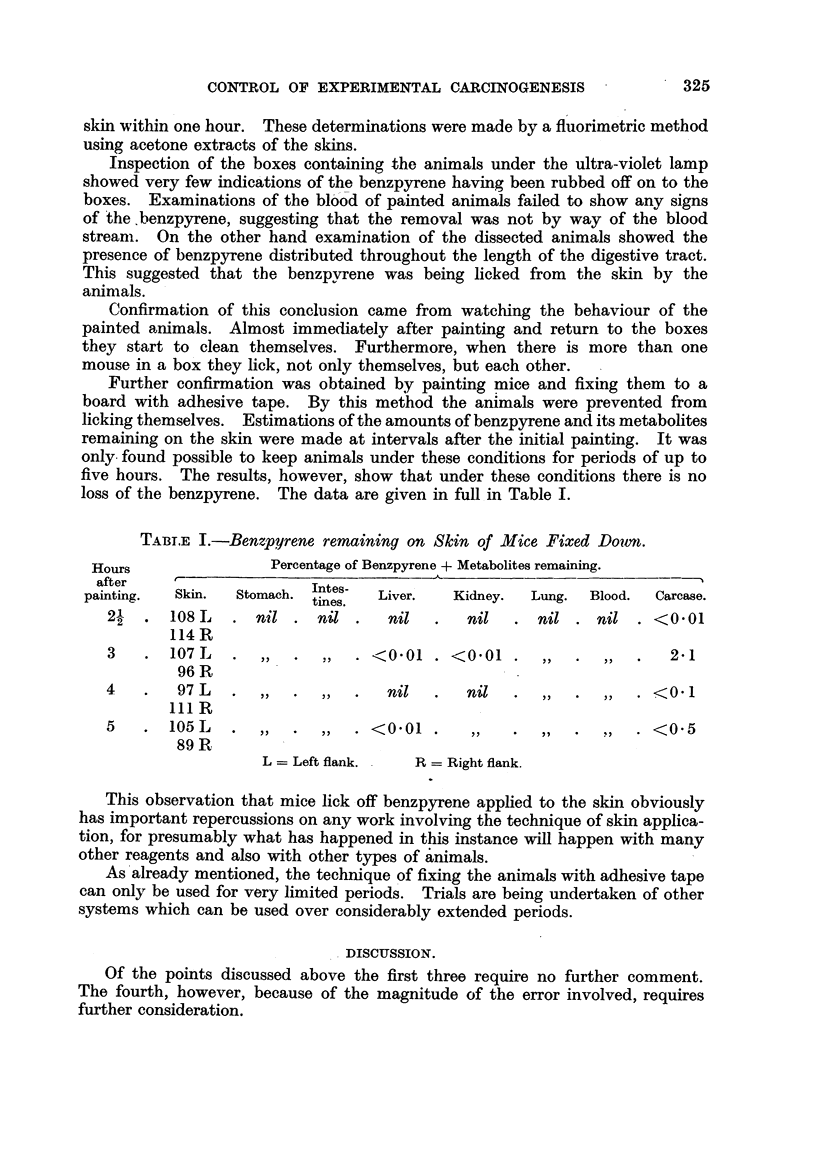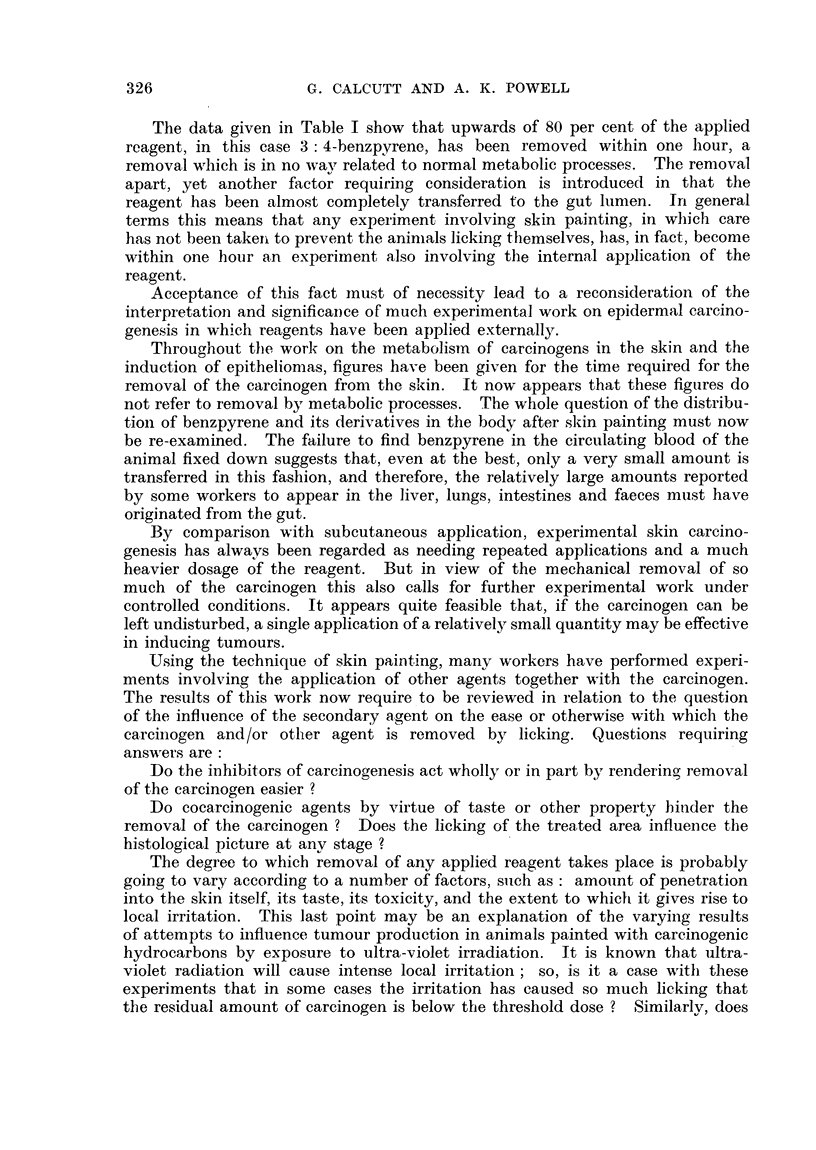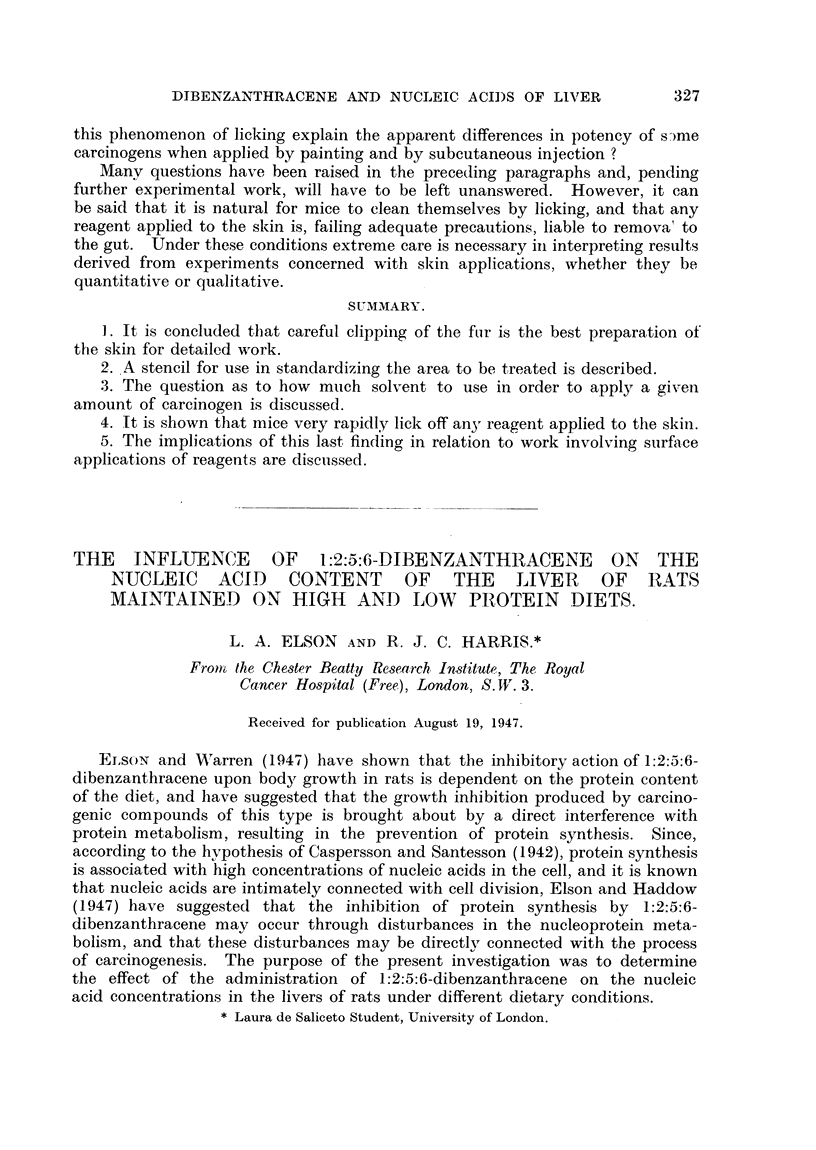# The Control of Some Factors Involved in Experimental Epidermal Carcinogenesis

**DOI:** 10.1038/bjc.1947.28

**Published:** 1947-09

**Authors:** G. Calcutt, A. K. Powell


### THE CONTROL OF SOME FACTORS INVOLVED IN EXPERIMENTAL EPIDERMAL CARCINOGENESIS.

G. CALCUTT AND A. K. POWELL.

*From the Department of Experimental Biology, Mount Vernon Hospital, and the Radium Institute, Northwood, Middlesex.*

Received for publication July 29, 1947.

PREPARATORY to commencing some detailed work on the metabolism of 3:4-benzpyrene in the skin of the mouse consideration was given to a number of experimental factors which appeared likely to affect the reproducibility of the results. As the processes it was desired to follow are primarily chemical, it was obvious that a careful standardization of technique had to be adopted so that the conditions governing any reactions were as nearly constant as possible.

Since the points considered and the implications to which they give rise have a bearing on various other types of work involving experimental skin carcinogenesis they are discussed in some detail below.

### FACTORS CONSIDERED.

1. In order to avoid injury to the mouse skin the use of depilatories was avoided, as they are liable to cause irritation and leave behind traces of unwanted chemicals. Instead, the fur was clipped away prior to application of the carcinogen. Surface examination of the treated area immediately after painting showed that considerable quantities of the reagent were adherent to the fur stubble in the form of crystals. In order to apply the carcinogen directly to the skin it is, therefore, essential that the fur be removed as completely as possible.

2. For quantitative work some standardization of the area to be treated was required. The method finally adopted was to apply the solution of benzpyrene through a stencil cut from sheet aluminium (16 gauge), with a hole of the desired size and shape. Even under these conditions examination by ultra-violet light showed that the painted areas varied considerably in size. Most of these variations could be ascribed to the spread of the solution under the edges of the stencil, but a certain amount was due to the flexibility of the mouse skin and the tension to which the skin was subjected in holding the animal. This problem was partially solved by the use of stencils with flanged edges (Fig. 1). With the down-turned edge against the skin and the stencil held firmly in position the area of skin is fairly well fixed and the edge helps to prevent the spread of the solution.

3. As it was desired to use a volatile solvent, acetone or benzene, for the application, the question of how much solvent to use in order to apply any given standard amount of the benzpyrene was examined. The selected amount of benzpyrene was 3 mg. The use of a small volume of solvent, 0·05 c.c., resulted in rapid drying and the deposition of the benzpyrene as a crust of crystals over the treated area. Considerable increase in the volume of the solvent gave trouble with the spread of the solution beyond the desired area. The volume finally selected for application was 0·1 c.c. This was found to give an even distribution over an area of 4 sq. cm. without the formation of masses of loose

Fig. 1.—Aluminium stencil.

Fig. 2.—The percentage of benzpyrene remaining on skin after painting.

crystals and without any appreciable danger of spreading beyond the desired area.

4. Bearing in mind the points raised above, a series of determinations of the amounts of benzpyrene left in the skin at various time intervals after painting were undertaken. The results are shown diagrammatically in Fig. 2. The rate of disappearance of the benzpyrene from the skin is found to be very rapid ; approximately 80–90 per cent of the benzpyrene having been removed from the skin within one hour. These determinations were made by a fluorimetric method using acetone extracts of the skins.

Inspection of the boxes containing the animals under the ultra-violet lamp showed very few indications of the benzpyrene having been rubbed off on to the boxes. Examinations of the blood of painted animals failed to show any signs of the benzpyrene, suggesting that the removal was not by way of the blood stream. On the other hand examination of the dissected animals showed the presence of benzpyrene distributed throughout the length of the digestive tract. This suggested that the benzpyrene was being licked from the skin by the animals.

Confirmation of this conclusion came from watching the behaviour of the painted animals. Almost immediately after painting and return to the boxes they start to clean themselves. Furthermore, when there is more than one mouse in a box they lick, not only themselves, but each other.

Further confirmation was obtained by painting mice and fixing them to a board with adhesive tape. By this method the animals were prevented from licking themselves. Estimations of the amounts of benzpyrene and its metabolites remaining on the skin were made at intervals after the initial painting. It was only found possible to keep animals under these conditions for periods of up to five hours. The results, however, show that under these conditions there is no loss of the benzpyrene. The data are given in full in Table I.

TABLE I.—*Benzpyrene remaining on Skin of Mice Fixed Down.*

| Hours after painting. | Percentage of Benzpyrene + Metabolites remaining. | | | | | | | |
|---|---|---|---|---|---|---|---|---|
| | Skin. | Stomach. | Intestines. | Liver. | Kidney. | Lung. | Blood. | Carcase. |
| $2\frac{1}{2}$ | 108 L<br>114 R | *nil* | *nil* | *nil* | *nil* | *nil* | *nil* | <0·01 |
| 3 | 107 L<br>96 R | ,, | ,, | <0·01 | <0·01 | ,, | ,, | 2·1 |
| 4 | 97 L<br>111 R | ,, | ,, | *nil* | *nil* | ,, | ,, | <0·1 |
| 5 | 105 L<br>89 R | ,, | ,, | <0·01 | ,, | ,, | ,, | <0·5 |

L = Left flank. R = Right flank.

This observation that mice lick off benzpyrene applied to the skin obviously has important repercussions on any work involving the technique of skin application, for presumably what has happened in this instance will happen with many other reagents and also with other types of animals.

As already mentioned, the technique of fixing the animals with adhesive tape can only be used for very limited periods. Trials are being undertaken of other systems which can be used over considerably extended periods.

### DISCUSSION.

Of the points discussed above the first three require no further comment. The fourth, however, because of the magnitude of the error involved, requires further consideration.

The data given in Table I show that upwards of 80 per cent of the applied reagent, in this case 3 : 4-benzpyrene, has been removed within one hour, a removal which is in no way related to normal metabolic processes. The removal apart, yet another factor requiring consideration is introduced in that the reagent has been almost completely transferred to the gut lumen. In general terms this means that any experiment involving skin painting, in which care has not been taken to prevent the animals licking themselves, has, in fact, become within one hour an experiment also involving the internal application of the reagent.

Acceptance of this fact must of necessity lead to a reconsideration of the interpretation and significance of much experimental work on epidermal carcinogenesis in which reagents have been applied externally.

Throughout the work on the metabolism of carcinogens in the skin and the induction of epitheliomas, figures have been given for the time required for the removal of the carcinogen from the skin. It now appears that these figures do not refer to removal by metabolic processes. The whole question of the distribution of benzpyrene and its derivatives in the body after skin painting must now be re-examined. The failure to find benzpyrene in the circulating blood of the animal fixed down suggests that, even at the best, only a very small amount is transferred in this fashion, and therefore, the relatively large amounts reported by some workers to appear in the liver, lungs, intestines and faeces must have originated from the gut.

By comparison with subcutaneous application, experimental skin carcinogenesis has always been regarded as needing repeated applications and a much heavier dosage of the reagent. But in view of the mechanical removal of so much of the carcinogen this also calls for further experimental work under controlled conditions. It appears quite feasible that, if the carcinogen can be left undisturbed, a single application of a relatively small quantity may be effective in inducing tumours.

Using the technique of skin painting, many workers have performed experiments involving the application of other agents together with the carcinogen. The results of this work now require to be reviewed in relation to the question of the influence of the secondary agent on the ease or otherwise with which the carcinogen and/or other agent is removed by licking. Questions requiring answers are :

Do the inhibitors of carcinogenesis act wholly or in part by rendering removal of the carcinogen easier ?

Do cocarcinogenic agents by virtue of taste or other property hinder the removal of the carcinogen ? Does the licking of the treated area influence the histological picture at any stage ?

The degree to which removal of any applied reagent takes place is probably going to vary according to a number of factors, such as : amount of penetration into the skin itself, its taste, its toxicity, and the extent to which it gives rise to local irritation. This last point may be an explanation of the varying results of attempts to influence tumour production in animals painted with carcinogenic hydrocarbons by exposure to ultra-violet irradiation. It is known that ultra-violet radiation will cause intense local irritation ; so, is it a case with these experiments that in some cases the irritation has caused so much licking that the residual amount of carcinogen is below the threshold dose ? Similarly, does this phenomenon of licking explain the apparent differences in potency of some carcinogens when applied by painting and by subcutaneous injection ?

Many questions have been raised in the preceding paragraphs and, pending further experimental work, will have to be left unanswered. However, it can be said that it is natural for mice to clean themselves by licking, and that any reagent applied to the skin is, failing adequate precautions, liable to remova' to the gut. Under these conditions extreme care is necessary in interpreting results derived from experiments concerned with skin applications, whether they be quantitative or qualitative.

SUMMARY.

1. It is concluded that careful clipping of the fur is the best preparation of the skin for detailed work.
2. A stencil for use in standardizing the area to be treated is described.
3. The question as to how much solvent to use in order to apply a given amount of carcinogen is discussed.
4. It is shown that mice very rapidly lick off any reagent applied to the skin.
5. The implications of this last finding in relation to work involving surface applications of reagents are discussed.